# Causal Transcription Regulatory Network Inference Using Enhancer Activity as a Causal Anchor

**DOI:** 10.3390/ijms19113609

**Published:** 2018-11-15

**Authors:** Deepti Vipin, Lingfei Wang, Guillaume Devailly, Tom Michoel, Anagha Joshi

**Affiliations:** 1 Division of Developmental Biology, The Roslin Institute, The University of Edinburgh, Easter Bush, Midlothian, EH25 9RG Scotland, UK; Deepti.Vipin@roslin.ed.ac.uk (D.V.); Guillaume.Devailly@roslin.ed.ac.uk (G.D.); 2 Division of Genetics and Genomics, The Roslin Institute, The University of Edinburgh, Easter Bush, Midlothian, EH25 9RG Scotland, UK; Lingfei.wang@roslin.ed.ac.uk (L.W.); Tom.Michoel@roslin.ed.ac.uk (T.M.); 3 Computational Biology Unit, Department of Informatics, University of Bergen, DataBlokk, 5th Floor, Thormohlensgt 55, N-5008 Bergen, Norway; 4 Computational Biology Unit, Department of Clinical Science, University of Bergen, DataBlokk, 5th Floor, Thormohlensgt 55, N-5008 Bergen, Norway

**Keywords:** transcription regulation, gene expression, causal inference, enhancer activity

## Abstract

Transcription control plays a crucial role in establishing a unique gene expression signature for each of the hundreds of mammalian cell types. Though gene expression data have been widely used to infer cellular regulatory networks, existing methods mainly infer correlations rather than causality. We developed statistical models and likelihood-ratio tests to infer causal gene regulatory networks using enhancer RNA (eRNA) expression information as a causal anchor and applied the framework to eRNA and transcript expression data from the FANTOM Consortium. Predicted causal targets of transcription factors (TFs) in mouse embryonic stem cells, macrophages and erythroblastic leukaemia overlapped significantly with experimentally-validated targets from ChIP-seq and perturbation data. We further improved the model by taking into account that some TFs might act in a quantitative, dosage-dependent manner, whereas others might act predominantly in a binary on/off fashion. We predicted TF targets from concerted variation of eRNA and TF and target promoter expression levels within a single cell type, as well as across multiple cell types. Importantly, TFs with high-confidence predictions were largely different between these two analyses, demonstrating that variability within a cell type is highly relevant for target prediction of cell type-specific factors. Finally, we generated a compendium of high-confidence TF targets across diverse human cell and tissue types.

## 1. Introduction

Despite having the same DNA, gene expression is unique to each cell type in the human body. Cell type-specific gene expression is controlled by short DNA sequences called enhancers, located distal to the transcription start site of a gene. Collaborative efforts such as the FANTOM [1] and Roadmap Epigenomics [2] projects have now successfully built enhancer and promoter repertoires across hundreds of human cell types, with an estimated 1.4% of the human genome associated with putative promoters and about 13% with putative enhancers. Enhancers physically interact with promoters to activate gene expression. Although the general rules governing these interactions (if any) remain poorly understood, experimental techniques such as chromosome conformation capture (3C, 4C) combined with next generation sequencing (Hi-C) [3], as well as computational methods based on correlations between histone modifications or DNase I hypersensitivity at enhancers with the expression of nearby promoters [4,5] are continually improving in predicting enhancer-promoter interactions. In contrast, understanding how the activation of one gene leads to the activation or repression of other genes, i.e., uncovering the structure of cell type, specific transcriptional regulatory networks, remains a major challenge. It is known that promoter expression levels of transcription factors (TFs) co-express and cluster together with promoters of functionally-related genes [6], but without any additional information, such associations are merely correlative and do not indicate a causal regulation by the TF.

Statistical causal inference aims to predict causal models where the manipulation of one variable (e.g., expression of gene A) alters the distribution of the other (e.g., expression of gene B), but not necessarily vice versa [7]. A key role in causal inference is played by causal anchors, variables that are known *a priori* to be causally upstream of others and that can be used to orient the direction of causality between other, relevant variables. A major application of this principle has been found in genetical genomics or systems genetics: genetic variations between individuals alter molecular and organismal phenotypes, but not vice versa, so these quantitative trait loci (QTL) can be used as causal anchors to determine the direction of causality between correlated traits from population-based data [8,9,10,11]. Such pairwise causal associations can then be assembled into causal gene networks to model how genetic variation at multiple loci collectively affect the status of molecular networks of genes, proteins, metabolites and downstream phenotypes [12].

Interestingly, several experiments have recently shown that enhancer regions can be transcribed to form short (around 1000 bp), non-coding, often bi-directional transcripts, called enhancer RNAs or eRNAs [13]. eRNAs have other distinguishing features, including nonpolyadenylated and unspliced tails, and tend to remain nuclear, rather than reaching the cytoplasm for translation. Although the functional role of some eRNAs has been studied in great detail to demonstrate that they can enhance or suppress enhancer activity by enhancer-promoter looping [14], the full repertoire of functional mechanisms of eRNAs remains to be understood. Nevertheless, the presence of eRNAs from a regulatory region is an indicator of enhancer activity [15], and eRNA expression has been successfully used to predict transcription factor activity [16]. Moreover, eRNA expression is correlated with and, crucially, *temporally precedes* the expression of target genes [17]. We therefore hypothesized that eRNA expression as a readout of enhancer activity could act as a causal anchor, opening new avenues to reconstruct causal gene regulatory networks.

To test this hypothesis, we developed novel statistical models and likelihood-ratio tests for using (continuous) eRNA expression data in causal inference, based on existing methods for discrete eQTL data, and implemented these in the Findr software [18]. We applied this new method to Cap Analysis of Gene Expression (CAGE) data generated by the FANTOM5 Consortium. Unlike RNA sequencing, CAGE allows sequencing of only the five prime ends of mRNAs, providing genome-wide transcription start site quantification at much lower sequencing depth (and therefore, lower sequencing cost per sample) than RNA-seq. The FANTOM Consortium has generated a unique resource of enhancer and promoter expression across hundreds of human and mouse cell types [6,17] and validated predictions using ChIP-seq and perturbation data. Our analysis of the FANTOM data showed that continuous eRNA expression values increased target prediction performance for some factors, while for other factors, a binarized presence or absence of the enhancer signal performed better. Leveraging this observation, we found that a data-driven approach to classify enhancer expression as either binary or continuous was sufficient to select the best target prediction method automatically, allowing parameter-free application of the method to organisms and cell types where validation data are not currently available.

## 2. Results

### 2.1. Development of a Causal Transcription Network Inference Framework

**Figure 1:**
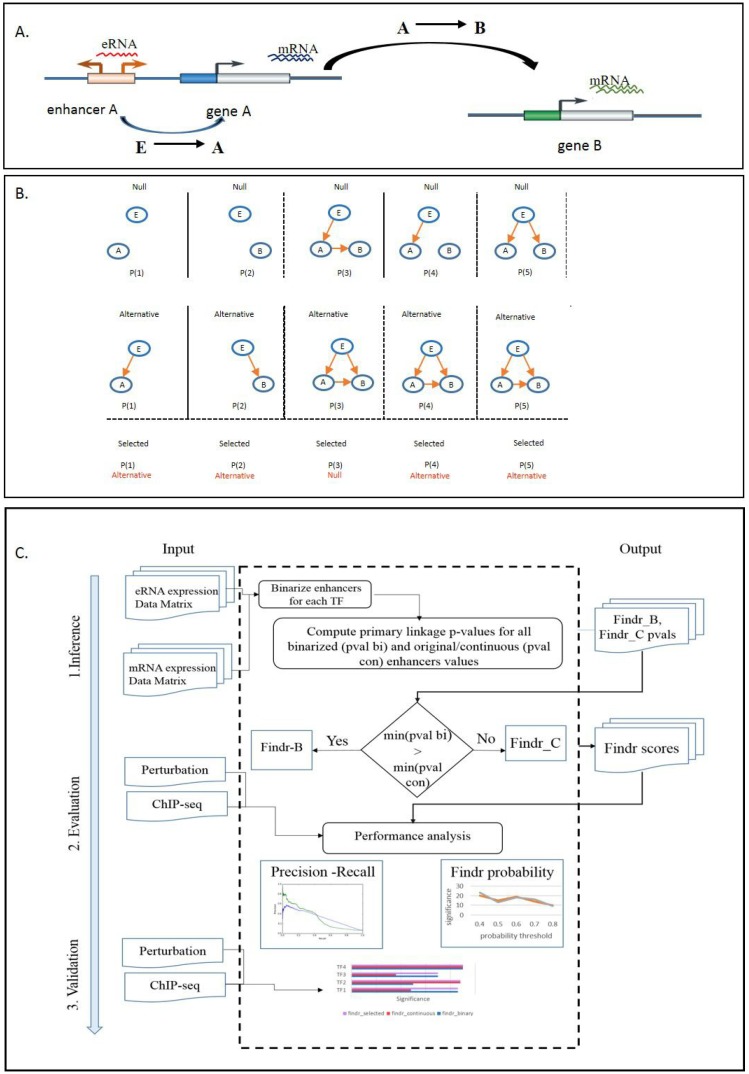
Overview of the Findr framework. (**A**) The schematic representation of causal gene regulatory network inference using enhancer activity as a causal anchor. (**B**) Five statistical tests used by Findr for causal inference. (**C**) Workflow of the Findr-A framework. eRNA, enhancer RNA; TF, transcription factor; B, binary; C, continuous.

We hypothesized that enhancer activity could be used as a causal anchor to predict causality between two co-expressed genes (Figure 1A). To this end, we used enhancer expression as a causal anchor to infer causal gene interactions, within the Findr framework [18]. Findr provides accurate and efficient inference of gene regulations using eQTLs as causal anchors by accounting for hidden confounding factors and weak regulations. This is achieved by performing and combining five likelihood ratio tests (Figure 1B), each of which consists of a null ($H_{\mathrm{null}}$) and an alternative ($H_{\mathrm{alt}}$) hypothesis, to support or reject the causal model E→A→B, where *E* is an enhancer in the regulatory region of gene *A*, and *B* is a putative target gene: primary linkage (E→A), secondary linkage (E→B), conditional independence (E→B only through *A*), *B*’s relevance (E→B or correlation between *A* and *B*) and excluding pleiotropy (partial correlation between *A* and *B* after conditioning on *E*). The log-likelihood ratios (LLRs) are computed for all possible targets of each gene and then converted into *p*-values and posterior probabilities [19] of the alternative hypothesis being true; see [18] for details.

We applied three treatments to enhancer expression data. First, we regarded enhancers as binary (on/off) variables, and after binarizing the data (see Methods), we used the existing Findr to predict TF targets directly. This approach will be referred to as Findr-B (“binary”). Second, we adapted all five tests in Findr to use continuous instead of discrete causal anchor data, and we used this method on (untransformed) eRNA data. This approach will be referred to as Findr-C (“continuous”). Third, to accommodate the co-existence of binary and continuous enhancers for different TFs within the same dataset, we developed an automatic adaptive method to treat each enhancer independently as binary or continuous, depending on the relative strength of the primary enhancer-TF linkage with either method. We call this approach Findr-A (“adaptive”). The workflow of the Findr framework is summarized in Figure 1C. We note that the implementation of the method is generic, i.e., it can be used by defining either eQTL genotypes or enhancer activity as causal anchors.

### 2.2. Causal Inference from Enhancer and Transcript/Gene Expression CAGE Data

To test our hypothesis that causal inference using enhancer expression as a causal anchor predicts true TF targets, we used CAGE data generated by the FANTOM Consortium across hundreds of cell types and tissues in human and mouse [6]. We used bi-directional expression in non-promoter regions as an indicator of likely enhancer activity in each CAGE sample [1]. Specifically, non-promoter regions with a similar number of sequence tags in both the forward and reverse direction were predicted as putative enhancer regions. Moreover, these regions also showed a high overlap (over 90 percent) with H3K4me1 modification. Importantly, CAGE data offer a powerful resource as each sample contains both enhancer and gene activity information. The ability to quantify enhancer expression (as a proxy for enhancer activity) and gene expression from the same sample is crucial for the ability to apply causal inference techniques. We first selected three mouse cell types (embryonic stem cells, macrophages and erythroblastic leukaemia) for systematic characterization, as these had more than 20 samples per cell type, with diverse treatments or time series. Furthermore, ChIP-seq and TF knock-out validation datasets were available for each of these cell types. In order to assign both promoter proximal, as well as promoter distal putative enhancers for each transcription factor, we selected predicted enhancers [1] within 50 kb of the transcription start site of each transcription factor in a cell type. This resulted in 109 enhancers for 48 transcription factors in ES cells, 55 enhancers for 8 transcription factors in macrophages and 5 enhancers for 4 transcription factors in erythroleukaemia, with an average of 3.8 enhancers per transcription factor across cell types.

**Figure 2:**
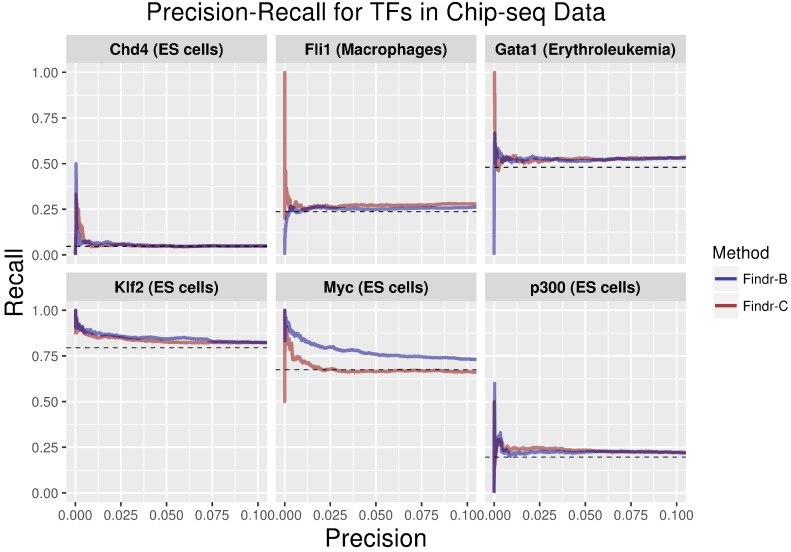
Recall-precision curves for target predictions by Findr-B and Findr-C using ChIP-seq. The dotted line represents the background or the the random classifier precision.

We inferred causal transcription factor-target interactions for each transcription factor using the enhancer element most strongly linked to each TF in each cell type. We predicted targets for 48 transcription factors with two methods, one using continuous enhancer data (“Findr-C”) and one using discretized, binary (on/off) enhancer data (“Findr-B”) (see Methods). The Findr software outputs a score representing the putative probability of a causal interaction for each transcription factor-target pair (see Methods). For both methods, the targets with a predicted probability of a causal interaction greater than 0.8 (see Methods) were validated using a compendium of ChIP-seq data [20], containing 78 factors in ES cells, 12 factors in macrophages and 17 factors in erythroblasts. Of these factors, 18 in ES cells, 7 in macrophages and 4 in erythroblasts had enhancer expression in CAGE data. We noted that the suitability of Findr-B or Findr-C for causal inference was dependent on the factor, i.e., using continuous enhancer data performed better for some factors (Figure 2: Myc,Klf2, Figure S1: Fcgr3), while on/off data performed better for others (Figure 2: Gata1, Fli1, Figure S2: Junb, Jarid2). Because the number of putative ChIP-seq targets for each factor varied widely across factors and cell types, from only 420 gene targets for JunD in erythroblasts to over 12,000 gene targets for ESRRB in ES cells, the background precision levels differed highly between factors (Figure 2).

**Figure 3:**
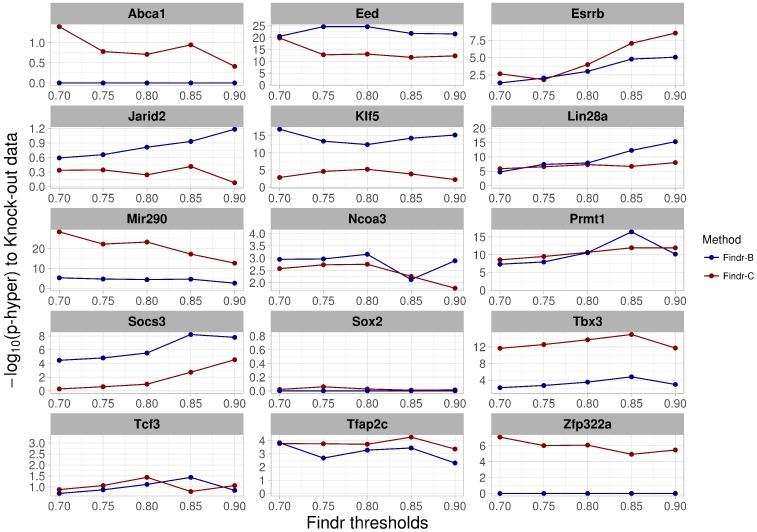
Robustness of Findr performance demonstrated by using different score thresholds.

We further tested whether these results were sensitive to the target probability prediction threshold. The enrichments of true positives were stable over a wide range around this threshold for both methods (Figure 3, Figure S2).

**Figure 4:**
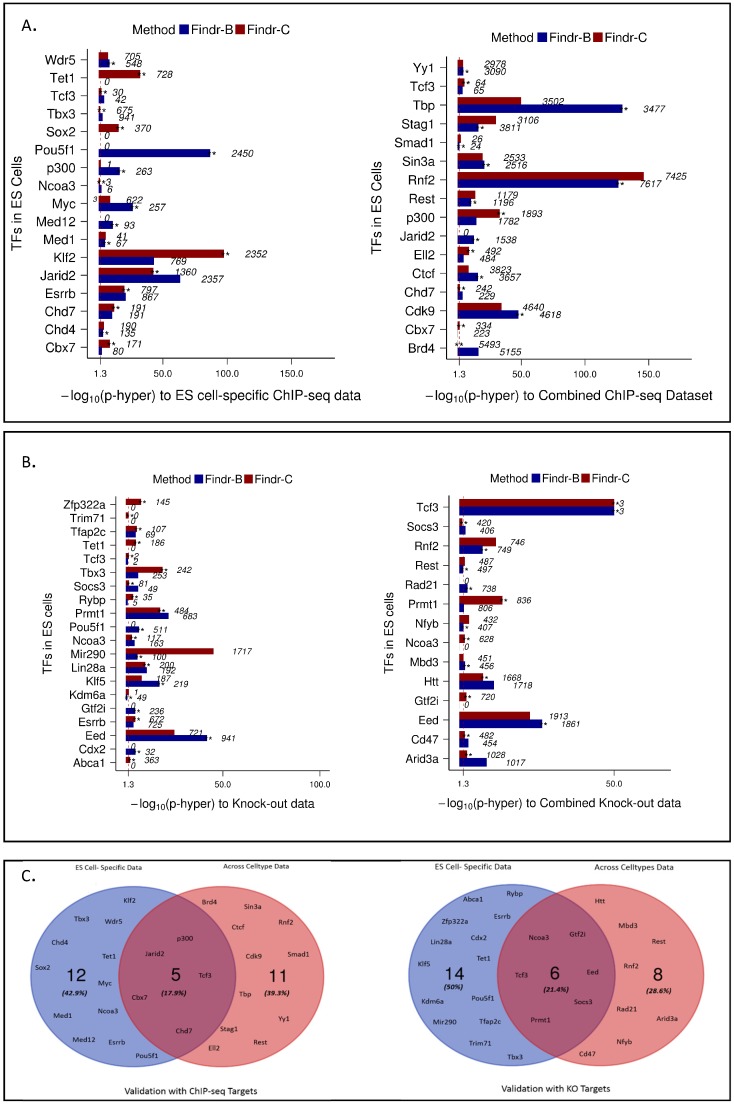
Comparison of Findr-adaptive (A) predictions using mouse ES cells and all cell types samples. (**A**) Bar plots representing enrichments for Findr-B, -C and -A predictions using ChIP-seq data as a validation dataset for ES cells (left) and all cell types (right). (**B**) Bar plots representing enrichments for Findr-B, -C and -A predictions using knock-out data as a validation dataset for ES cells (left) and all cell types (right). (**C**) Overlap of factors between ES and all cell types using ChIP-seq (left) and knock-out (right) as validation datasets.

Transcription factor binding inferred using ChIP-seq data is thought to be mostly opportunistic and therefore might not provide direct clues about the functional targets of the factor [21]. We therefore collected perturbation data from publications, specifically expression data after knock-out or knock-down (KO) of a factor. We generated differentially-expressed gene lists for 85 factors in ES cells and 11 factors in macrophages (see Methods). Using these gene lists as known targets, we evaluated the predictions of both methods. This confirmed the factor-specific suitability of either the Findr-B or Findr-C method (Figure 4, Figure S3).

The evaluation so far used the default combination of likelihood-ratio tests in Findr (Figure 1B, Tests 2, 4 and 5; see the Methods for details), which was previously shown to perform better than the traditional causal inference method, which relies on a conditional independence test (Figure 1B, Test 3), by accounting for hidden confounding due to common upstream regulatory factors between a TF and its targets [18]. To test whether this remains true for CAGE data, we implemented all five tests in both Findr-B and Findr-C. We found that the new test combination improved predictive power for the CAGE data in all three mouse cell types compared to the traditional test combination, similar to the previous results on eQTL data. We have therefore set it as the default choice for the Findr tool.

### 2.3. Development and Validation of an Adaptive Model-Selection Approach for Causal Inference Using Discretized or Continuous Data

As the optimal prediction performance depended on a factor-specific choice between discretizing enhancer expression data or not, we investigated if this decision could be made in a data-driven, adaptive approach (called “Findr-adaptive” or “Findr-A”; see Figure 1C), in the absence of validation data. In short, Findr-A selects for each TF among all its candidate enhancers, both continuous and binary, the one with the strongest primary linkage to the TF’s expression and then uses that enhancer and its corresponding method (Findr-B or Findr-C) to predict downstream targets for that TF (see the Methods for details). This adaptive approach was indeed able to select the best performing method for most of the factors (Figure 4A,B, Findr-A selection marked by double stars).

We further performed functional enrichment analysis of gene target sets predicted by Findr-A. 1119 targets of JunB in macrophages were enriched for ‘LPS signalling pathway’ ($p<10^{-8}$) and ‘RNA binding’ ($p<10^{-8}$) (Tables S1 and S2). The macrophage CAGE samples indeed measured the response to LPS signalling and JunB is known to be a delayed response gene, attenuating transcriptional activity of immediate early genes and RNA binding proteins, specifically terminating translation of mRNAs induced by immediate early genes [22]. 131 targets for Cbx7 in ES cells (Figure 3) were enriched for ‘regulation of transcription from RNA polymerase II promoter’ ($p<10^{-10}$), and included several developmental genes such as Hox family proteins (Tables S3 and S4). This enrichment was much stronger than for the ChIP bound targets of Cbx7 ($p<10^{-5}$). Cbx7 is a part of the PRC complex, which binds predominantly at bivalent chromatin at promoters of transcription regulators in ES cells [23].

### 2.4. Perturbations within and across Cell Types Provide Causal Targets for a Distinct Set of Transcription Factors

We noted that most factors performed better using binary enhancer expression values and wondered if this might be due to the limited enhancer expression data available for each cell type. We therefore explored whether the enhancer activity across perturbations within cell type was comparable to variation of enhancer activity across different cell and tissue types. To test this, we used the FANTOM5 CAGE data containing over 1000 samples across 360 distinct mouse cell types and tissues, called “all-data”. Findr-C indeed performed marginally better on all-data rather than cell type-specific data. Importantly, all-data and cell type (ES)-specific data resulted in causal targets for a distinct set of transcription factors. In particular, variation within a cell type was more informative for causal target predictions of cell type-specific factors. For example, the targets of key pluripotency factors Sox2 and Esrrb [24] were enriched in ES-data, but not all-data (Figure 4A,B).

There were only five common factors with causal targets predicted using both all-data and ES-specific data that were validated by ChIP-seq data and only six common factors validated by KO data (Figure 4C). Interestingly, the target genes predicted from ES-data and all-data for the same factor overlapped significantly. For example, 72% of Eed predicted targets using ES-data overlapped with Eed predicted targets using all-data.

### 2.5. Multiple Enhancers of the Same Factor Have a Highly Correlated Expression

Mammalian genes are controlled by multiple enhancers. We investigated the stability of inference outcome under different choices of enhancers as causal anchors. For all factors for which Findr-A predicted targets in ES cells that overlapped significantly with perturbation data (Figure 4), we predicted additional target sets using other available enhancers, resulting in target sets for 76 enhancer-transcription factor pairs for 46 unique transcription factors.

The hierarchical clustering of transcription factor-enhancer pairs based on these target sets clustered mostly by transcription factors, indicating that the expression of multiple enhancers contains highly redundant information about the activity of the associated transcription factor (Figure S5). Reassuringly, factors known to form regulatory complexes, including Max and Mxi1 or Runx1 and Smad1, also clustered together, i.e., shared predicted causal targets (Figure S5).

To investigate whether combining multiple enhancers was more informative for determining causal targets, we compared two integrative methods against the predictions of taking individual enhancers. Firstly, we used the median expression level of all the putative enhancers for each transcription factor as a ‘meta-enhancer’ in Findr-A (Figure S4A). Secondly, we calculated the first principal component of the binary target prediction matrix for all enhancer of a TF in order to ‘average’ predictions (Figure S4B). However, we did not observe any significant overall improvement in performance using either method.

### 2.6. Causal Inference Using CAGE Expression Data across Human Cell Types

**Figure 5:**
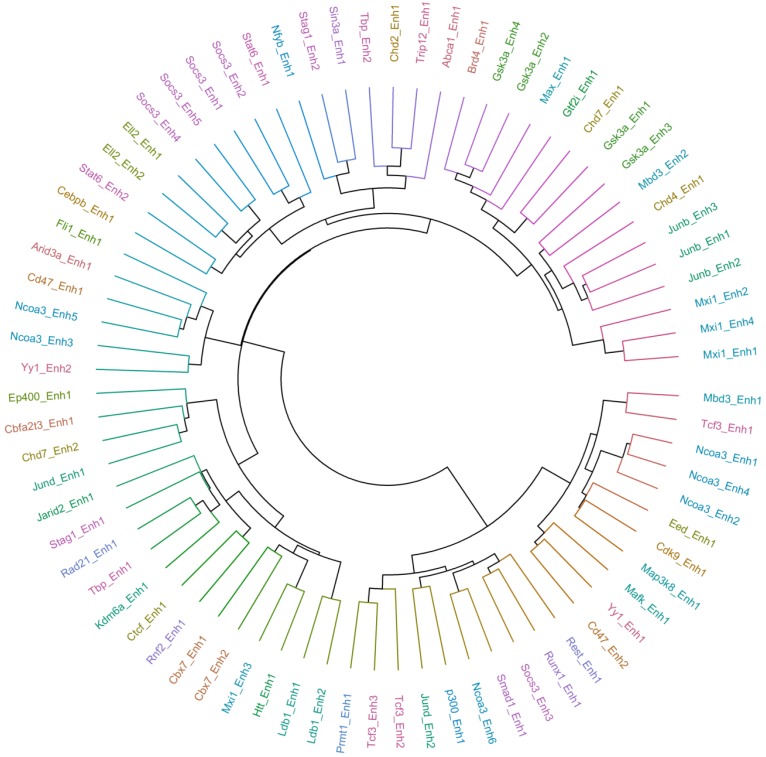
Hierarchical representation of similarities between transcription factor-target sets predicted using Findr-A causal inference on the FANTOM5 human dataset.

Finally, we inferred causal interactions between transcription regulators and targets using CAGE enhancer and TSS expression data in humans. Specifically, we inferred causal interactions for 20 transcription factors (with eRNA expression) using Findr-A (see Methods). We firstly validated the predicted interactions using a database of experimentally-validated regulatory interactions in human [25] and noted a statistically-significant overlap between the predicted and experimentally-validated gene sets ($p<10^{-5}$). Figure 5 represents the hierarchical clustering of the predicted top 200 interactions for each factor. We noted that multiple enhancers of the same factor predicted highly overlapping targets for that factor. Moreover, the factors involved in biologically-related processes shared predicted causal targets. For example, two members of the SMAD family, SMAD3 and SMAD6, as well as BCOR and SIN3A involved in histone deacetylase activity showed a high overlap of predicted targets.

## 3. Discussion

We explored the utility of eRNA expression as a causal anchor to predict transcription regulatory networks, by leveraging the observation that eRNAs mark the activity of regulatory regions. Previous studies support this notion, as eRNA expression has been shown to precede the expression of its effector gene temporally [17] and to correlate strongly with active regulatory regions across cell types [15]. We therefore developed a novel statistical framework to infer causal gene networks (Findr-A), by extending the Findr software for causal inference using eQTL data [18].

We demonstrated the applicability of Findr-A by predicting causal interactions from CAGE data generated through the FANTOM Consortium and validating them with ChIP-seq and perturbation data for three mouse cell types, as well as on the entire FANTOM5 data. Notably, different factors were enriched for within cell type analysis as compared to across cell types. The causal regulatory network of cell type-specific factors (e.g., Sox2, Esrrb in ES cells) could be inferred only using expression variation within a cell type and not across cell types. Due to the limited availability of validation data, a more comprehensive assessment was not possible.

The current approach can be extended in several aspects in the future. Firstly, Findr assumes equal (or no) relations between all sample pairs [18], which hold for the majority of eQTL datasets. By accounting for heterogeneous sample relationships, such as biological and/or technical replicates, time series or population structure, we may be able to reconstruct more accurate networks. Secondly, the assumption that eRNAs act as causal anchors is only approximately true, because their activity ultimately is regulated by other regulatory factors, i.e., the assumption that they are a priori causally upstream of correlated TF-gene pairs will not hold for all genes. Because eRNAs are temporally expressed before their direct target genes, we hypothesize that explicit modelling of gene expression dynamics in the Findr framework will allow detecting and correcting for such feedback loops. Thirdly, eRNAs are expressed at relatively low levels, and therefore susceptible to noise, and a reliable eRNA signal was available for only a limited number of known transcription factors in mouse or human. Generating deep sequencing data for CAGE, utilizing GRO-seq or epigenetic or transcription factor ChIP-seq data to estimate enhancer activity could be possible ways to get around this.

In this work, we have used a very basic approach for associating enhancers with their likely promoters based on their mutual proximity, as associating enhancers with promoters was not our main focus. This could be refined by using existing data generated by methods such as chromatin confirmation capture or by integrating multiple genomic features into a statistical predictor for associating enhancers with promoters [5].

Finally, we used ChIP-seq and KO data for validation, because these datasets were available for the same cell types as the expression data in mouse. These data are far from ideal as ground-truth sets, as ChIP-seq data tend to be noisy and differential expression in transcription factor knock-outs could include indirect effects. Moreover, the targets predicted by ChIP-seq and KO experiments show a very poor overlap between them. For example, a large-scale study where 59 TFs were knocked down in a human lymphoblastoid cell line (GM12878) concluded that only a small subset of genes bound by a factor were differentially expressed following knock-down of that factor [21]. It is therefore important to note that there is no universal agreement on ground-truth TF-target regulatory networks for validation, including networks obtained from literature mining. For example, there are only 151 common TF-target interactions among four literature-curated databases, which together comprise more than 10,000 interactions [26]. As shown, our method predicts targets for many TFs w.r.t. to one ground-truth as well or better than the other ground-truth, reaffirming our hypothesis that eRNA data can be used to infer TF targets.

In conclusion, we have demonstrated that enhancer activity can be used to infer causal gene regulatory networks. We foresee this approach to be of high value in the context of human medicine, by combining genetic, epigenetic and transcriptomic information across individuals to unravel causal disease networks.

## 4. Materials and Methods

### 4.1. Datasets

- We used Cap Analysis of Gene Expression (CAGE) data (TPM expression values) from the FANTOM5 Consortium for enhancer and transcription start sites (TSS) in mouse embryonic stem (ES) cells (36 experiments), macrophages (224 experiments) and erythroblastic leukaemia (52 experiments) [6,17]. We also selected 1036 samples from all cell types and tissues in mouse.
- We use ChIP-seq data from the Codex Consortium [20]. Data available for 78 TFs in mouse ES cells, 12 TFs in macrophages and 17 TFs in erythroleukaemia cells were used.
- For validation using knock-out data, we have collected differentially-expressed gene lists after perturbation of factors from published studies in mouse and gene lists after overexpression of factors in mouse ES cells from [27].
- We obtained CAGE data (TPM expression values) from the FANTOM5 Consortium for enhancer and TSSs for human cell types and tissues [6,17]. The enhancer regions identified using bi-directional expression were obtained from [1]. The FANTOM5 Consortium has provided data for 1826 samples from which we selected 360 samples from all cell types and tissues, one sample for each cell and tissue type with the highest sequencing depth (removing technical and biological replicates).

### 4.2. Data Processing

- CAGE data were processed to clear unannotated and non-expressed genes, and expression levels were log-transformed. Genes with a TPM value of 1 or more in at least one sample were considered expressed. Only enhancers expressed in more than one third of experiments were retained.
- For each TF, we selected the promoter with the highest median expression level as the promoter for that TF.
- For each TF, all enhancers within 50 kb of the TF promoter region were detected using the GenomicRanges package in Bioconductor [28] and considered as candidate causal anchor enhancers for that TF. The Findr framework (described below in detail) includes a “primary linkage” step such that targets are only predicted for TFs with significantly correlated eRNAs.
- Enhancer data were binarized by setting all experiments with zero read count to zero and all others to one.
- For the ChIP-seq data, genes with a TF binding site within 1 kb of their TSS were defined as targets for that TF.
- For the knock-out and over-expression data, genes with differential expression *q*-value<0.05 were defined as targets for the TF.

### 4.3. Likelihood Ratio Tests with Continuous Causal Anchor Data

Given a causal relation E→A→B to test, where *E* is a (continuous) enhancer for TF *A* and *B* is a putative target gene, with their expression data samples 1,…,n annotated in subscripts, we first convert each continuous variable into a standard normal distribution by rank. Each variable is modelled as a normal distribution with the mean linearly and additively dependent on its regulators in the five tests below (illustrated in Figure 1B).

1. **Primary linkage test**: The primary linkage test verifies that the enhancer *E* regulates the regulator gene *A*. Its null and alternative hypotheses are:
  $$H_{\mathrm{null}}^{\left(1\right)}\equiv E\;A,\;H_{\mathrm{alt}}^{\left(1\right)}\equiv E\to A.$$
  The log likelihood ratio (LLR) and its null distribution are identical to the correlation test in [18]. Therefore, the LLR is:
  $${\mathrm{LLR}}^{\left(1\right)}=-\frac{n}{2}\ln\left(1-{\hat{\rho }}_{EA}^{2}\right),$$
  where:
  $${\hat{\rho }}_{XY}\equiv \frac{1}{n}\sum_{i=1}^{n}X_{i}Y_{i}\;.$$
  Its null distribution is:
  $${\mathrm{LLR}}_{\mathrm{null}}^{\left(1\right)}/n∼D\left(1,n-2\right).$$
  The probability density function (PDF) for $z∼D(k_{1},k_{2})$ is defined as: for z>0,
  $$p\left(z|k_{1},k_{2}\right)=\frac{2}{B(k_{1}/2,k_{2}/2)}{\left(1-e^{-2z}\right)}^{\left(k_{1}/2-1\right)}e^{-k_{2}z},$$
  and for z≤0, $p(z|k_{1},k_{2})=0$, where B(a,b) is the Beta function.
2. **Secondary linkage test**: The secondary linkage test verifies that the enhancer *E* regulates the target gene *B*. The LLR and its null distribution are identical to those of the primary linkage test, except by replacing *A* with *B*.
3. **Conditional independence test**: The conditional independence test verifies that *E* and *B* become independent after conditioning on *A*, with its null and alternative hypotheses as:
  $$\begin{array}{ccc} H_{\mathrm{null}}^{\left(3\right)} & \equiv & E\to A\to B,\\ H_{\mathrm{alt}}^{\left(3\right)} & \equiv & B\leftarrow E\to A\wedge (A\;\mathrm{correlates}\;\mathrm{with}\;B). \end{array}$$
  Correlated genes are modelled as having a multi-variant normal distribution, whose mean linearly depends on their regulator gene. Therefore,
  $$\begin{array}{ccc} {\mathrm{LLR}}^{\left(3\right)} & = & -\frac{n}{2}\ln\left(\left(1-{\hat{\rho }}_{EA}^{2}\right)\left(1-{\hat{\rho }}_{EB}^{2}\right)-{\left({\hat{\rho }}_{AB}-{\hat{\rho }}_{EA}{\hat{\rho }}_{EB}\right)}^{2}\right)\\ & & +\frac{n}{2}\ln\left(1-{\hat{\rho }}_{EA}^{2}\right)+\frac{n}{2}\ln\left(1-{\hat{\rho }}_{AB}^{2}\right). \end{array}$$
  Following the same definition of the null data, their null distribution is:
  $${\mathrm{LLR}}_{\mathrm{null}}^{\left(3\right)}/n∼D\left(1,n-3\right).$$
4. **Relevance test**: The relevance test verifies that *B* is regulated by either *E* or *A*. Its hypotheses are:
  $$\begin{array}{ccc} H_{\mathrm{null}}^{\left(4\right)} & \equiv & E\to A\;B,\\ H_{\mathrm{alt}}^{\left(4\right)} & \equiv & E\to A\wedge E\to B\leftarrow A. \end{array}$$
  Similarly,
  $$\begin{array}{ccc} {\mathrm{LLR}}^{\left(4\right)} & = & -\frac{n}{2}\ln\left(\left(1-{\hat{\rho }}_{EA}^{2}\right)\left(1-{\hat{\rho }}_{EB}^{2}\right)-{\left({\hat{\rho }}_{AB}-{\hat{\rho }}_{EA}{\hat{\rho }}_{EB}\right)}^{2}\right)\\ & & +\frac{n}{2}\ln\left(1-{\hat{\rho }}_{EA}^{2}\right). \end{array}$$
  $${\mathrm{LLR}}_{\mathrm{null}}^{\left(4\right)}/n∼D\left(2,n-3\right).$$
5. **Controlled test**: The controlled test verifies that *E* regulates *B* through *A*, partially or fully, with the hypotheses as:
  $$\begin{array}{ccc} H_{\mathrm{null}}^{\left(5\right)} & \equiv & B\leftarrow E\to A,\\ H_{\mathrm{alt}}^{\left(5\right)} & \equiv & B\leftarrow E\to A\wedge A\to B. \end{array}$$
  Its LLR is
  $$\begin{array}{ccc} {\mathrm{LLR}}^{\left(5\right)} & = & -\frac{n}{2}\ln\left(\left(1-{\hat{\rho }}_{EA}^{2}\right)\left(1-{\hat{\rho }}_{EB}^{2}\right)-{\left({\hat{\rho }}_{AB}-{\hat{\rho }}_{EA}{\hat{\rho }}_{EB}\right)}^{2}\right)\\ & & +\frac{n}{2}\ln\left(1-{\hat{\rho }}_{EA}^{2}\right)+\frac{n}{2}\ln\left(1-{\hat{\rho }}_{EB}^{2}\right), \end{array}$$
  with the null distribution:
  $${\mathrm{LLR}}_{\mathrm{null}}^{\left(5\right)}/n∼D\left(1,n-3\right).$$

The LLR and its null distribution then allow one to compute the *p*-values and the posterior probabilities of the null and alternative hypotheses separately for each subtest, as detailed in [18].

### 4.4. Findr-B and Findr-C

In [18], it was shown that a combined causal inference test performs best in terms of sensitivity and specificity for recovering true regulatory interactions, using both real and simulated test data. The combined test score is:

$$P=\frac{1}{2}\left(P_{2}P_{5}+P_{4}\right)$$

where $P_{i}$ is the posterior probability for subtest *i*.

The Findr-B method returns this combined *p*-value using the original Findr on binarized enhancer data. Findr-C does the same using the new tests on continuous enhancer data.

### 4.5. Adaptive Method Findr-A

Given a set of TFs and for every TF, a set of candidate causal anchor enhancers, the adaptive Findr-A method performs the following, for each TF *A* (Figure 1C):

1. Compute the primary linkage test *p*-value for all candidate enhancers of *A*, both continuous and binarized.
2. Find the enhancer *E* with the lowest *p*-value overall.
3. If the lowest *p*-value occurred for a binarized enhancer, use Findr-B for TF *A* with *E* as its causal anchor, else use Findr-C.

Findr-A, -B and -C have been implemented in the Findr software, available at https://github.com/lingfeiwang/findr.

### 4.6. Validation Methods

For the purpose of evaluation, we calculated the Findr-B and Findr-C scores for all TF-gene combinations. Genes with scores exceeding a threshold of 0.8 of Findr score were considered as predicted targets for each TF. Precision-recall curves were calculated using the the “PRROC” package. We used the FDR corrected (BH procedure) hyper-geometric test for enrichment analysis, where the overlap with respect to known targets from ChIP-seq and knock-out data in the same cell type was tested, and the resulting *p*-values were used to compare the performance of the two methods.

## Supplementary Materials

The following are available online at http://www.mdpi.com/1422-0067/19/11/3609/s1.
