# Supplementary material for: Causal Transcription Regulatory Network Inference Using Enhancer Activity as a Causal Anchor
Source: Int J Mol Sci. 2018 Nov 15;19(11):3609. doi: 10.3390/ijms19113609 (PMC6274755; doi:10.3390/ijms19113609)
Supplement: Supplementary file 1 [file ijms-19-03609-s001.pdf]

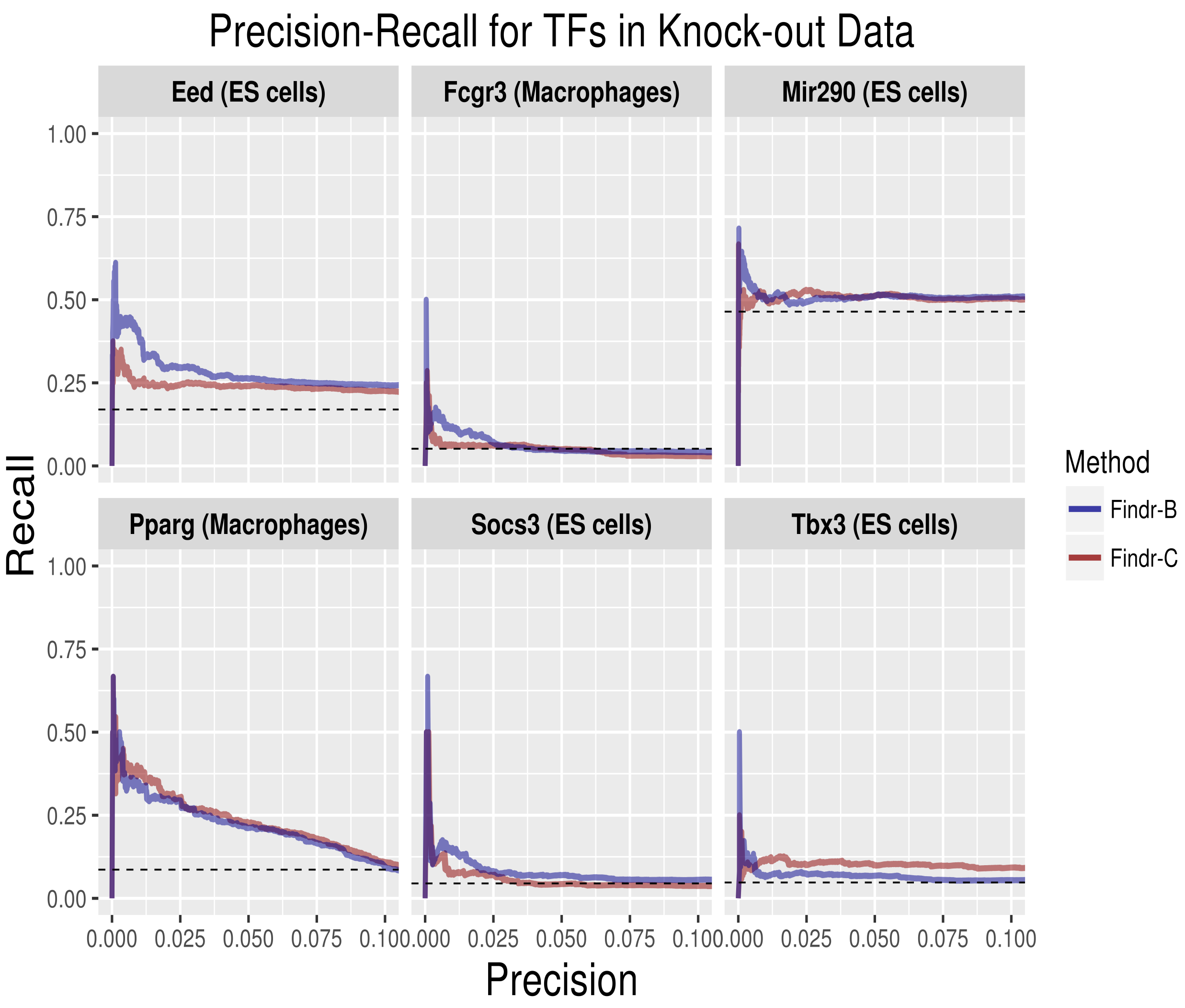

Figure S1: Recall-precision curves for targets predicted by Findr-B and findr-C using perturbation data in ES calls and macrophages. The dotted line represnets the background or random precision.

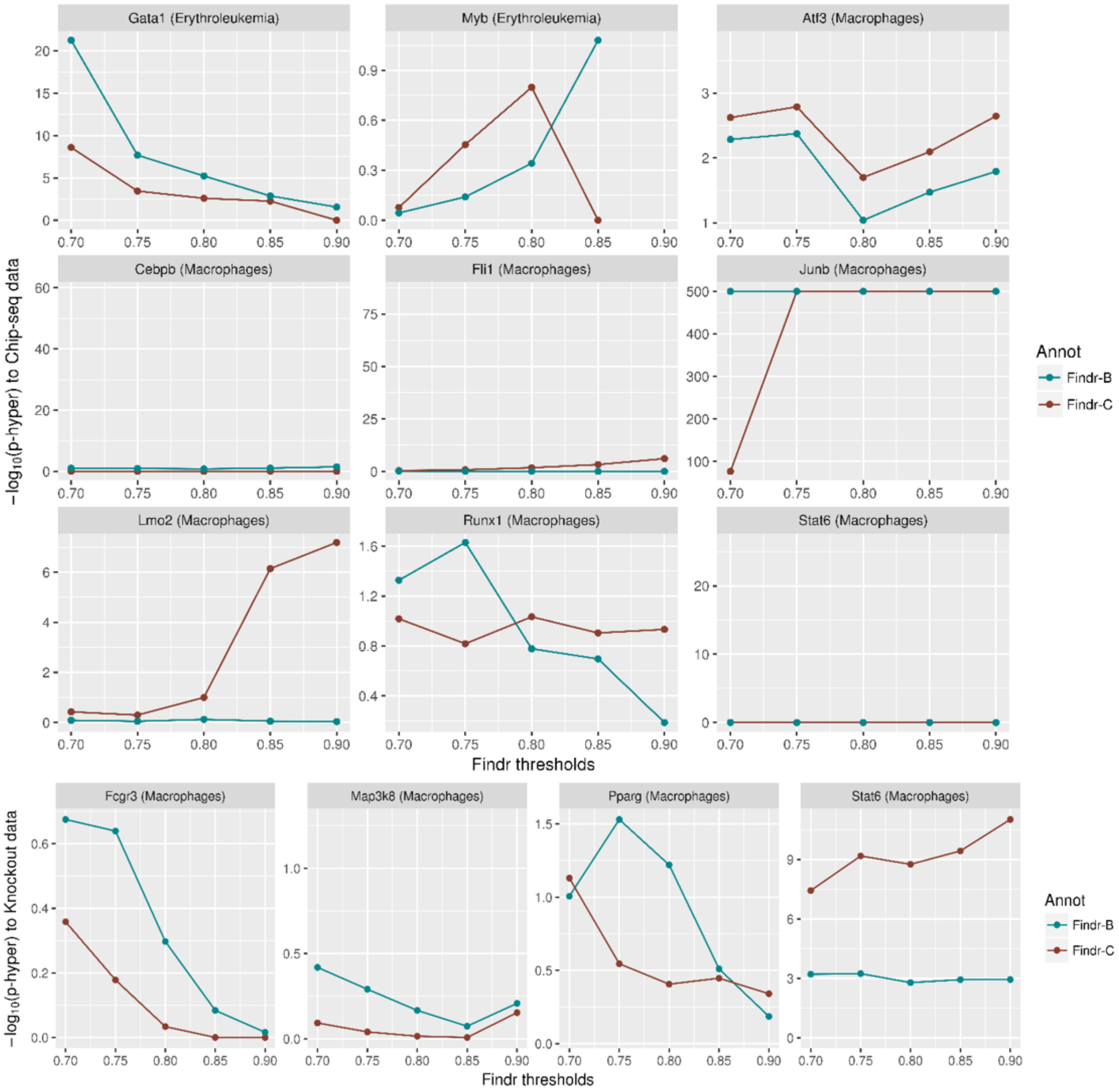

Figure S2: Robustness of Findr performance demonstrated by using different score thresholds in macrophages and erythroleukemia cells

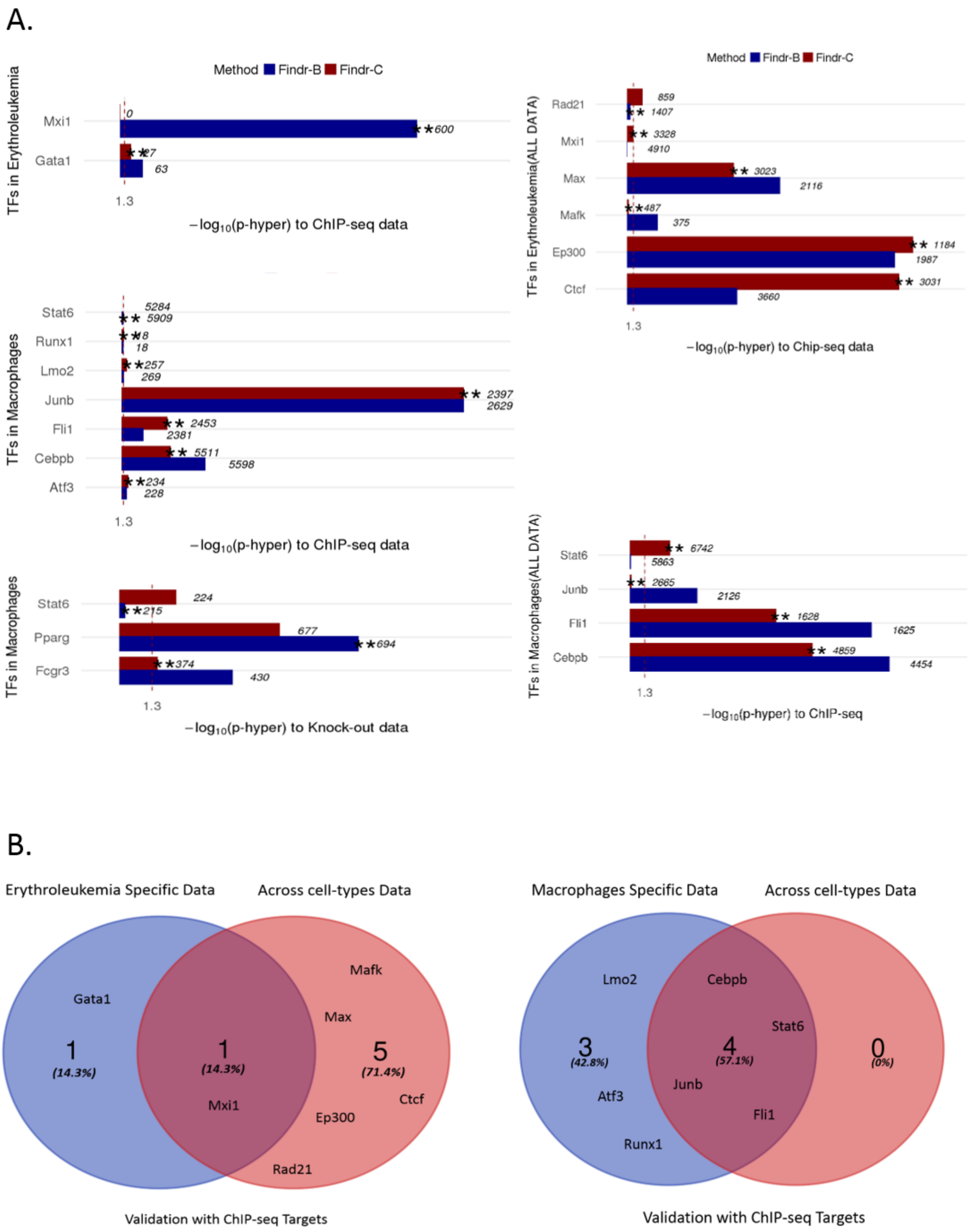

A.
Method Findr-B Findr-C
TFs in Erythroleukemia
Mxi1
Gata1
0
600
7
63
1.3
−log10(p-hyper) to ChIP-seq data
TFs in Macrophages
Stat6
Runx1
Lmo2
Junb
Fli1
Cebpb
Atf3
5284
5909
8
18
257
269
2397
2629
2453
2381
5511
5598
234
228
1.3
−log10(p-hyper) to ChIP-seq data
TFs in Macrophages
Stat6
Pparg
Fcgr3
224
215
677
694
374
430
1.3
−log10(p-hyper) to Knock-out data
Method Findr-B Findr-C
TFs in Erythroleukemia(ALL DATA)
Rad21
Mxi1
Max
Mafk
Ep300
Ctcf
859
1407
3328
4910
3023
2116
487
375
1184
1987
3031
3660
1.3
−log10(p-hyper) to Chip-seq data
TFs in Macrophages(ALL DATA)
Stat6
Junb
Fli1
Cebpb
6742
5863
2665
2126
1628
1625
4859
4454
1.3
−log10(p-hyper) to ChIP-seq
B.
Erythroleukemia Specific Data
Across cell-types Data
Gata1
1
(14.3%)
1
(14.3%)
Mxi1
Mafk
Max
5
(71.4%)
Ctcf
Ep300
Rad21
Validation with ChIP-seq Targets
Macrophages Specific Data
Across cell-types Data
Lmo2
3
(42.8%)
Atf3
Runx1
Cebpb
Stat6
4
(57.1%)
Junb
Fli1
0
(0%)
Validation with ChIP-seq Targets

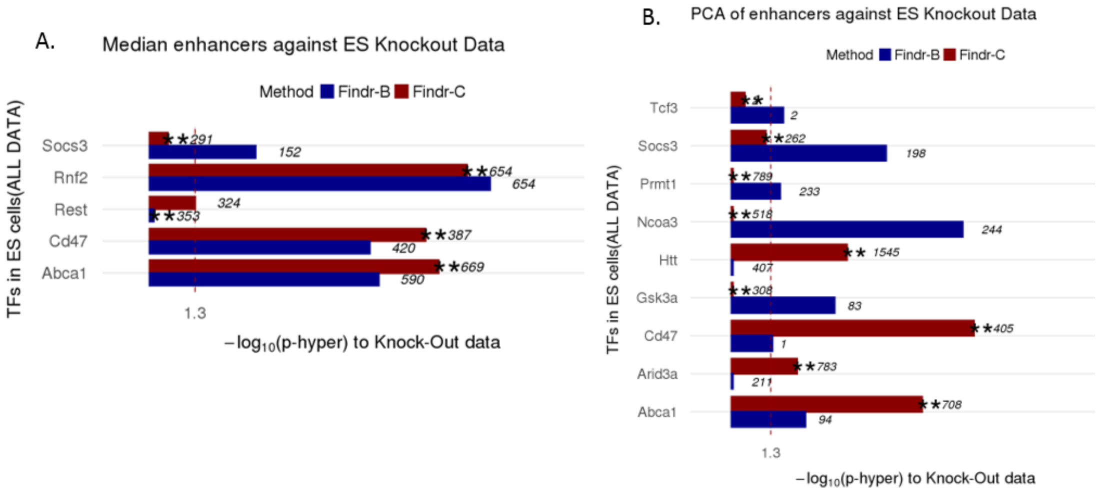

Figure S4: Performance of Findr when taking the median expression levels of enhancers(A) and first PC of enhancer expression (B).

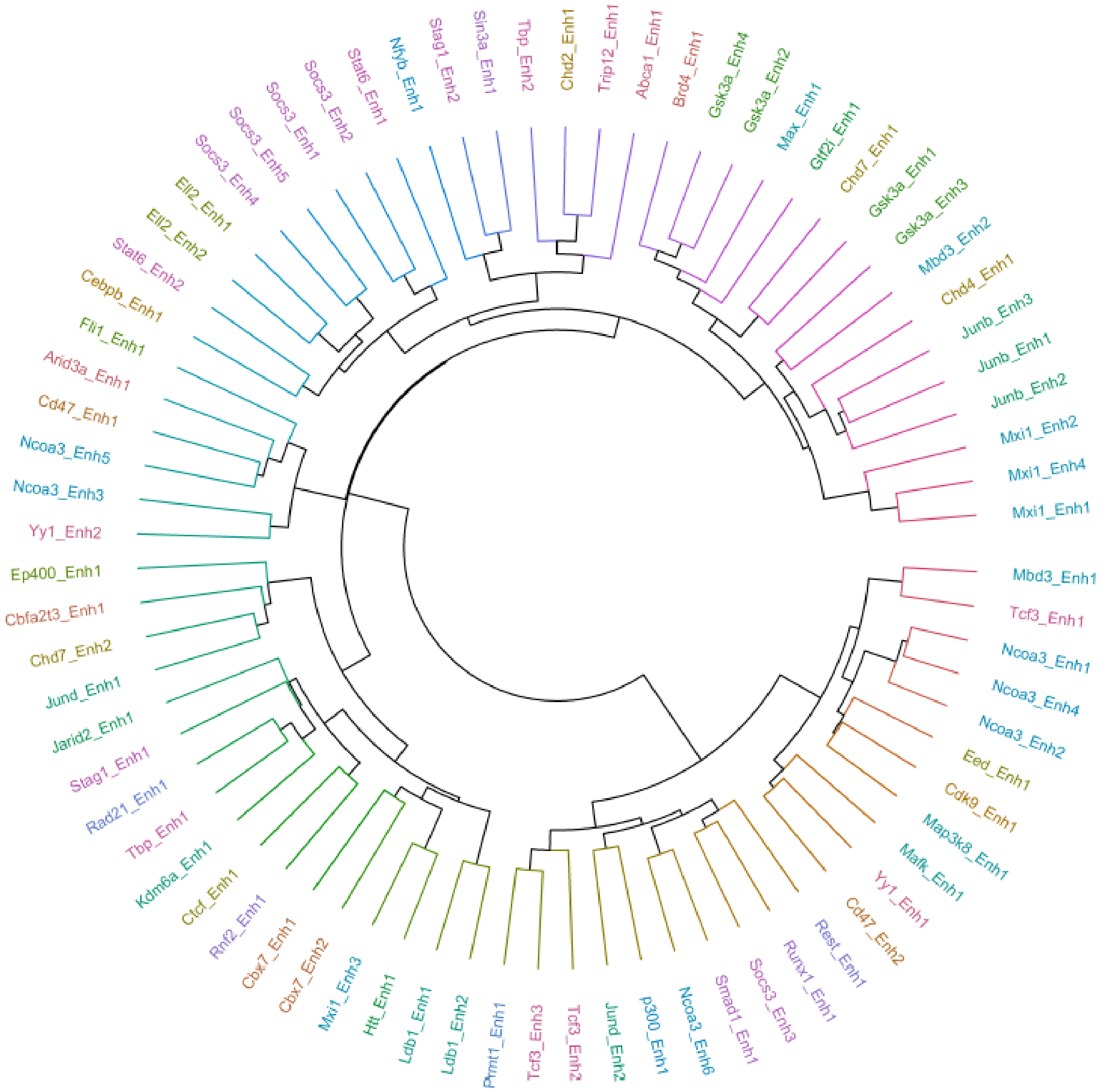

Figure S5: Circular plot of hierarchical clustering of overlap of targets predicted using multiple enhancers for the same factor in mouse ES cells

Index	Name	P-value	Adjusted p-value	Z-score	Combined score
1	positive regulation of transcription from RNA polymerase II promoter	0.000005725	0.001048	-7.62	91.98
2	lipopolysaccharide-mediated signaling pathway	1.284e-12	9.409e-9	-2.88	78.95
3	neutrophil degranulation	5.727e-7	0.0003238	-5.21	74.88
4	cellular response to lipopolysaccharide	8.063e-10	0.000002954	-3.33	69.70
5	positive regulation of NF-kappaB import into nucleus	1.948e-8	0.00004757	-3.75	66.54
6	I-kappaB phosphorylation	7.979e-7	0.0003654	-4.58	64.29
7	mRNA catabolic process	7.015e-8	0.00008565	-3.48	57.26
8	positive regulation of NF-kappaB transcription factor activity by ER overload response	3.155e-8	0.00005778	-3.29	56.78
9	negative regulation of cysteine-type endopeptidase activity involved in apoptotic process	0.00001126	0.001594	-4.70	53.52
10	positive regulation of I-kappaB phosphorylation	0.000001792	0.0005251	-4.03	53.34

Table s1: GO enrichment (biological process) for 1119 predicted JUNB targets in mouse macrophages

Table s2: GO enrichment (molecular function) for 1119 predicted JUNB targets in mouse macrophages

Index	Name	P-value	Adjusted p-value	Z-score	Combined score
1	telomerase RNA binding	1.748e-10	3.491e-8	-3.83	85.96
2	mRNA binding	3.160e-10	3.491e-8	-3.85	84.17
3	miRNA binding	3.165e-10	3.491e-8	-3.78	82.64
4	translation factor activity, RNA binding	4.525e-10	3.491e-8	-3.82	82.19
5	single-stranded RNA binding	6.124e-10	3.491e-8	-3.68	78.06
6	GU repeat RNA binding	5.145e-10	3.491e-8	-3.64	77.89
7	alpha-aminoacyl-tRNA binding	5.145e-10	3.491e-8	-3.64	77.77
8	21U-RNA binding	5.145e-10	3.491e-8	-3.63	77.58

Index	Name	P-value	Adjusted p-value	Z-score	Combined score
1	positive regulation of transcription from RNA polymerase II promoter	4.367e-16	1.231e-12	-7.77	274.82
2	negative regulation of transcription from RNA polymerase II promoter	2.103e-13	2.963e-10	-7.23	210.97
3	positive regulation of transcription, DNA-templated	5.636e-11	8.672e-9	-7.21	170.24
4	positive regulation of transcription from RNA polymerase II promoter involved in neuron differentiation	2.951e-11	6.827e-9	-6.74	163.44
5	positive regulation of ethanol catabolic process by positive regulation of transcription from RNA polymerase II promoter	2.793e-10	1.306e-8	-6.43	141.37
6	positive regulation of termination of RNA polymerase II transcription	2.793e-10	1.306e-8	-6.43	141.36
7	positive regulation of transcription from RNA polymerase II promoter involved in meiotic cell cycle	2.793e-10	1.306e-8	-6.41	141.08
8	positive regulation of mating type switching by positive regulation of transcription from RNA polymerase II promoter	2.793e-10	1.306e-8	-6.41	141.03
9	positive regulation of filamentous growth of a population of unicellular organisms in response to starvation by positive regulation of transcription from RNA polymerase II promoter	2.793e-10	1.306e-8	-6.41	140.97
10	positive regulation of transcription from RNA polymerase II promoter by calcium-mediated signaling	2.793e-10	1.306e-8	-6.40	140.88

Index	Name	P-value	Adjusted p-value	Z-score	Combined score
9	ribonuclease P RNA binding	5.145e-10	3.491e-8	-3.62	77.48
10	7S RNA binding	5.413e-10	3.491e-8	-3.63	77.47

Table S3: GO enrichment (biological process) for 131 predicted CBX7 targets in mouse ES cells

Table S4: GO enrichment (biological process) for top 131 CBX7 bound genes (ChIP-seq, GEO accession number GSM1041373) in mouse ES cells

Index	Name	P-value	Adjusted p-value	Z-score	Combined score
1	positive regulation of transcription from RNA polymerase II promoter	8.875e-9	0.000009860	-7.77	143.99
2	negative regulation of transcription from RNA polymerase II promoter	5.496e-9	0.000009860	-7.23	137.53
3	positive regulation of DNA repair by positive regulation of transcription from RNA polymerase II promoter	3.510e-7	0.00003696	-6.62	98.33
4	positive regulation of transcription from RNA polymerase II promoter involved in neuron differentiation	6.530e-7	0.00003696	-6.68	95.11
5	regulation of cellular ketone metabolic process by positive regulation of transcription from RNA polymerase II promoter	0.000001100	0.00003696	-6.41	87.92
6	positive regulation of snRNA transcription from RNA polymerase II promoter	0.000001100	0.00003696	-6.40	87.86
7	positive regulation of pseudohyphal growth by positive regulation of transcription from RNA polymerase II promoter	0.000001100	0.00003696	-6.40	87.85
8	positive regulation of purine nucleotide biosynthetic process by positive regulation of transcription from RNA polymerase II promoter	0.000001100	0.00003696	-6.40	87.84
9	positive regulation of starch catabolic process by positive regulation of transcription from RNA polymerase II promoter	0.000001100	0.00003696	-6.39	87.67
10	positive regulation of ribosomal protein gene transcription from RNA polymerase II promoter	0.000001100	0.00003696	-6.39	87.66
